# Should all pyloromyotomies for infantile hypertrophic pyloric stenosis be performed by pediatric surgeons?

**DOI:** 10.1007/s00383-026-06376-9

**Published:** 2026-04-11

**Authors:** Jordan Perkins, Joe Rodriguez, Simin Park, Richard Herman, Shin Miyata

**Affiliations:** Department of Pediatric Surgery, SSM Cardinal Glennon Children’s Hospital, St. Louis University School of Medicine, 1465 S. Grand Blvd, St. Louis, MO 63104 USA

**Keywords:** Pyloromyotomy, Pyloromyotomies, Infantile hypertrophic pyloric stenosis

## Abstract

**Purpose:**

Pyloromyotomies for infantile hypertrophic pyloric stenosis in academic centers are generally performed by pediatric surgeons (PS), while in non-specialized centers these are performed by general surgeons (GS). This cross-sectional study aims to address the paucity of data comparing the safety between PS and GS when performing a pyloromyotomy within NSQIP-P participating institutions.

**Methods:**

Data from 2012 to 2020 was obtained from the ACS-National Surgical Quality Improvement Program Pediatric (NSQIP-P) database. All patients who underwent pyloromyotomy by GS or PS were included. Patients who underwent other concurrent procedures were excluded. Demographics and postoperative outcomes were compared. Bivariate analyses and multivariable logistic regression were performed with a P-value < 0.05 being considered statistically significant.

**Results:**

A total of 18,453 pyloromyotomies were identified. Of these, 731 (4%) of cases were performed by GS and 17,722 (96%) by PS. The analysis indicated that several patient characteristics (weight, race, ASA class, comorbidities) and intra- and post-operative characteristics (operative length and hospital length of stay) were significantly different between groups. After adjusting for known risk factors, post-operative complications, re-admission rate, mortality and rate of re-operation were statistically similar between GS and PS. General surgeons were more likely to perform the operation via an open approach compared to pediatric surgeons (Adjusted OR 1.24 for Open vs. Laparoscopic, 95% CI 1.04–1.49). No significant difference was found in conversion rates (Adjusted OR 1.45 for Conversion to Open vs. Laparoscopic, 95% CI 0.68–3.08).

**Conclusion:**

Our findings suggest no difference in 30-day outcomes within NSQIP-P pediatric-focused institutions. However, these results may not generalize to community or non-participating hospitals due to potential misclassification of surgeon specialty and selection bias.

**Level of Evidence:**

III.

## Introduction

Infantile hypertrophic pyloric stenosis (IHPS) remains one of the most common surgical diseases of early infancy, usually presenting within the first three months of life with a 4:1 to 6:1 male predominance [1]. Classically it presents as projectile, nonbilious emesis after feeds that can lead to dehydration and associated electrolyte derangements. Diagnosis is largely made based on history and an ultrasound with a muscle thickness greater than 3 mm and length of the area greater than 15 mm [1]. The treatment for IHPS is a pyloromyotomy, a well-established surgical repair first performed in 1912 by Conrad Ramstedt [2]. Although the approach has shifted from open to laparoscopic, the principles remain the same: incision over the hypertrophied pylorus down to submucosa, spreading of muscle fibers, and leak test for mucosal perforation.

It is well established that surgical outcomes are tied to hospital and surgeon case volumes [3–6]. In pediatric surgery, this relationship has been explored, but data comparing general surgeons (GS) and pediatric surgeons (PS) for pyloromyotomy remain limited [7, 8]. The aim of this study is to evaluate if pyloromyotomies for IHPS have similar postoperative outcomes when performed by GS and PS within NSQIP-P participating institutions.

## Methods

### Study population and data collection 2012–2020

Data were obtained from the National Surgical Quality Improvement Program, Pediatric (NSQIP-P), a database maintained by the American College of Surgeons and American Pediatric Surgical Association. The database consists of de-identified information on demographics, pre-operative, intra-operative, post-operative, and discharge variables on patients under 18 years of age undergoing major surgical procedures that are selected by the NSQIP 8-day cycle systematic sampling system. Patient outcomes are assessed for 30 days following the procedure. As of 2015, 80 sites participate in the NSQIP-P.

All patients were restricted to infants under 1 year of age (median age 35 days, all confirmed < 18 years with no adult cases identified). Diagnosis codes included ICD-10 K31.1 (adult hypertrophic pyloric stenosis) and Q40.0 (congenital hypertrophic pyloric stenosis), as well as ICD-9 750.5 (congenital pyloric stenosis) and 537.0 (acquired pyloric stenosis). Procedure codes were limited to CPT 43,520 (pyloromyotomy) and CPT 43,659 (unlisted laparoscopic procedure of stomach, used as proxy for laparoscopic pyloromyotomy due to the absence of a specific CPT code for laparoscopic approach in the NSQIP-P dataset during the study period).

Exclusions included patients with concurrent procedures (e.g., gastrostomy tube placement, circumcision, pyloroplasty CPT 43800), surgeon specialty other than general surgery or pediatric surgery, and any additional CPT codes not related to isolated pyloromyotomy. Surgeon specialty was classified according to the surgeon-of-record designation in the NSQIP-P administrative coding as either general surgeon (GS) or pediatric surgeon (PS). Potential misclassification bias exists, as some pediatric surgeons may be recorded as general surgeons in pediatric hospitals; this is discussed in the Limitations section.

### Statistical analysis

The primary outcome was 30-day postoperative complications (composite of surgical site infection, wound disruption, reoperation, readmission, bleeding requiring transfusion, and death). Secondary outcomes included individual complications, readmission (overall and surgery-related), reoperation, mortality, operative approach, conversion rate, operative time, and length of stay.

Categorical variables were reported as frequencies and percentages for the entire population as well as the subgroups within the population. Similarly, continuous variables were reported as medians with associated first and third quartiles [IQR] for the entire population as well as the subgroups within the population. Categorical variables were compared using Chi-square test. Prior to conducting analysis of continuous variables, the Kolmogorov-Smirnov test was used to evaluate normality which showed violation with the normality assumption. For this reason, continuous variables were compared with the Kruskal-Wallis test. P values were reported for both continuous and categorical variables, with a significant threshold being a p-value less than 0.05.

Multivariable logistic regression was performed to adjust for known confounders: age (days), sex, ASA class, weight (kg), and key comorbidities (prematurity, pulmonary comorbidity, cardiac comorbidity, weight loss/FTT, bleeding/hematologic disorder). Weight was converted to kg (1 lb = 0.4536 kg) for SI unit compliance.

Statistical analysis was performed using SAS software, Version 9.4 of the SAS System for Windows. Descriptive statistics were presented as frequencies and proportions for categorical variables. Complete case analysis was used; no imputation was performed due to low missing rates in key variables (operative approach 7.9%, race 16.8%, ASA classification 0.21%, prematurity 1.6%). No propensity score matching or hospital-level clustering adjustment was performed due to data constraints; multivariable regression was used to adjust for confounders instead. Additional time-trend analyses were performed by dividing the study period into 2-year increments to explore changes in surgeon specialty and operative approach over time.

## Results

### Patient demographics

**Fig. 1:**
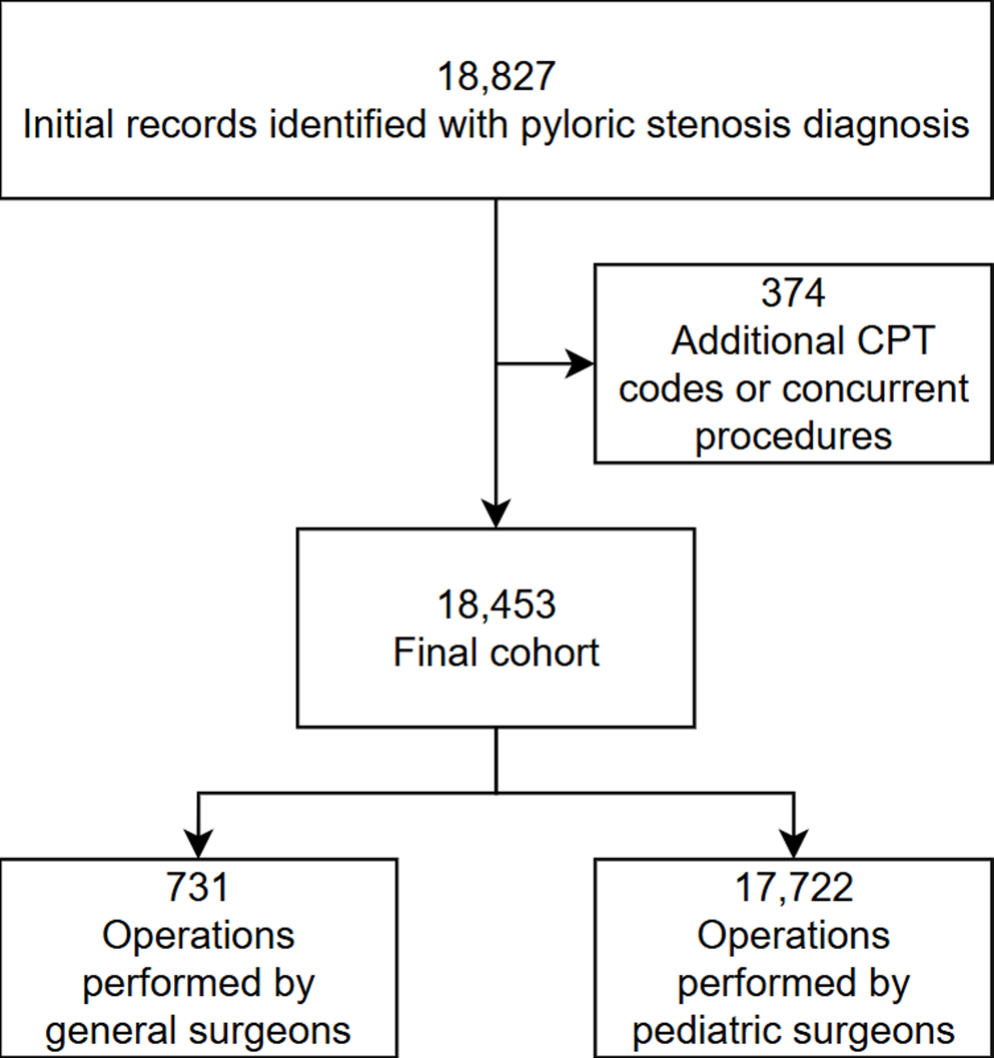
Study Population Flowchart. This diagram demonstrates how the study population was obtained after exclusion including the subgroup breakdown

A total of 18,827 patient records were initially identified from the NSQIP-P database with a diagnosis of pyloric stenosis. Any patient with a CPT code listed other than 43,520 (pyloromyotomy) or 43,659 (unlisted laparoscopic procedure of stomach) were excluded. This included any patients who had additional procedures listed such as g-tubes or circumcisions. After exclusion, the final study sample size included 18,453 records, with 731 of the operations performed by GS and 17,722 performed by PS (Fig. 1). Table 1 describes and compares the demographics of the total population as well as by the operating physician specialty. Overall, the population was found to be largely male with a median age of 35 days. Patients operated on by PS were found to weigh more on average but were also more likely to have comorbidities like prematurity, pulmonary or failure to thrive/weight loss comorbidity. No difference was observed in liver, biliary, pancreas, renal, bleeding/hematologic, cardiac conditions, or multiple comorbidities, though several of these conditions were subject to low numbers limiting analysis. Additionally, statistically significant differences were observed between the American Society of Anesthesiologists (ASA) classification and race between the two subgroups.

**Table 1:** Patient Demographics with Stratification by Operating Physician Specialty

Male	Total *n* = 18,453	General Surgeon *n* = 731	Pediatric Surgeon *n* = 17,722	*p*-value
	15,336 (83.1)	614 (84.0)	14,722 (83.1)	0.514
Age (days)	35 [27, 47]	35 [28, 44]	35 [27, 47]	0.570
Weight (kg)	3.9 [3.4, 4.4]	3.8 [3.4, 4.3]	3.9 [3.4, 4.4]	< 0.05
Race				< 0.05
White	13,509 (73.2)	608 (83.2)	12,901 (72.8)	
Black	1588 (8.6)	47 (6.4)	1541 (8.7)	
Asian	181 (1.0)	7 (1.0)	174 (1.0)	
Other	85 (0.5)	1 (0.1)	84 (0.5)	
ASA Classification				< 0.05
I	3885 (21.1)	155 (21.2)	3730 (21.1)	
II	11,443 (62.0)	487 (66.6)	10,956 (61.8)	
III	2993 (16.2)	86 (11.8)	2907 (16.4)	
IV	92 (0.5)	3 (0.4)	89 (0.5)	
V	1 (0.0)	0 (0.0)	1 (0.0)	
Premature Birth	1597 (8.7)	48 (6.6)	1549 (8.7)	< 0.05
Pulmonary Comorbidity	299 (1.6)	5 (0.7)	294 (1.7)	< 0.05
LBP Comorbidity	9 (0.05)	1 (0.1)	8 (0.1)	0.271
Renal Comorbidity	4 (0.02)	0 (0.0)	4 (0.0)	0.685
Weight loss/FTT Comorbidity	193 (1.1)	2 (0.3)	191 (1.1)	< 0.05
Cardiac Comorbidity	673 (3.6)	17 (2.3)	656 (3.7)	0.241
Minor	430 (2.3)	11 (1.5)	419 (2.4)	
Major	220 (1.2)	6 (0.8)	214 (1.2)	
Severe	23 (0.1)	0 (0.0)	23 (0.1)	
Multiple Comorbidities	129 (0.7)	3 (0.4)	126 (0.7)	0.339
Bleeding/Hematologic Disorder	121 (0.7)	4 (0.6)	117 (0.7)	0.711

Variables reported as frequency (percentage) or median [first quartile, third quartile] where appropriate. Percentages may not add up to 100% due to missing or not reported information. Categorical variables were compared using chi-square test of independence. Continuous variables were first evaluated for normality using Kolmogorov-Smirnov test to evaluate for normality and compared using the Kruskal-Wallis test

*FTT* failure to thrive, *LBP* liver biliary pancreas

### Operative demographics

Operative data from each specialty were also compared (Table 2). PS were found to have a longer operative time (median of 27 compared to 25 for GS, p = < 0.05). A statistical, but not clinically significant, difference was also observed in the days from operation to discharge (median of 1 for both GS and PS). The distribution of operative approach showed GS more likely to use open (75.0% vs. 67.3% for PS, *p* = 0.052). Rates of post-operative complications, readmission, and death were low, with no differences between the groups.

**Table 2:** Operative Demographics with Stratification by Operating Physician Specialty

	Total *n* = 18,453	General surgeon *n* = 731	Pediatric surgeon *n* = 17,722	*p*-value
Operative approach				0.052
Laparoscopic	4276 (23.2)	154 (21.1)	4122 (23.3)	
Open	12,475 (67.6)	548 (75.0)	11,927 (67.3)	
Attempted laparoscopic	4514 (24.5)	161(22.0)	4353(24.6)	
Conversion to open*	238 (5.3)	7 (4.3)	231 (5.3)	
Operation Length (minutes)	27 [20, 35]	22 [16, 29]	27 [20. 35]	< 0.05
Length of Stay (days)	2 [1, 3]	2 [1, 3]	2 [1, 3]	0.533
Operation to Discharge (days)	1 [1, 2]	1 [1, 1]	1 [1, 2]	< 0.05
Surgical Infection	247 (1.3)	9 (1.2)	238 (1.3)	0.797
Superficial	169 (0.9)	7 (1.0)	162 (0.9)	0.904
Deep	12 (0.1)	1 (0.1)	11 (0.1)	0.437
Organ Space	15 (0.1)	1 (0.1)	14 (0.1)	0.591
Wound Disruption	56 (0.3)	2 (0.3)	54 (0.3)	0.881
Reoperation	204 (1.1)	4 (0.6)	200 (1.1)	0.141
Readmission	561 (3.0)	23 (3.2)	538 (3.0)	0.865
Related to Surgery	307 (1.7)	15 (2.1)	292 (1.7)	0.402
Bleeding Requiring Transfusion	30 (0.2)	1 (0.1)	29 (0.2)	0.860
Death	7 (0.0)	1 (0.1)	6 (0.0)	0.161

Variables reported as frequency (percentage) or median [first quartile, third quartile] where appropriate. Percentages may not add up to 100% due to missing or not reported information. Categorical variables were compared using chi-square test of independence. Continuous variables were first evaluated for normality using Kolmogorov-Smirnov test to evaluate for normality and compared using the Kruskal-Wallis test. *Conversion to open among attempted laparoscopic cases

### Adjusted analysis

Logistic regression was utilized to further compare operative outcomes (Table 3). The only statistically significant difference found was comparing operative approach which found that GS were more likely to perform the operation via open compared to laparoscopic (Adjusted OR 1.24, 95% CI 1.04–1.49). No significant difference was found in conversion to open procedure (Adjusted OR 1.45, 95% CI 0.68–3.08). Odds of post-operative complications (infection, re-operation, readmission, bleeding requiring transfusion, and death) were found to be similar between the groups. The difference in length of operation was again observed using linear and multivariate analysis which demonstrated that GS had, on average, a shorter operative time compared to PS (p = < 0.05, Table 4). Time-trend analysis by 2-year periods demonstrated that the proportion of cases performed by general surgeons remained low (ranging from 2.5% to 6.0%) and the use of the laparoscopic approach showed only modest variation over the study period (Table 5).

**Table 3:** Crude and Adjusted Odds Ratios From Logistic Regression Comparing General Surgeons to Pediatric Surgeon

	Crude OR (95% CI)	Adjusted OR* (95% CI)
Operative Approach		
Open vs. Laparoscopic	1.23 (1.03–1.48)	1.24 (1.04–1.49)
Conversion to Open vs. Laparoscopic	1.52 (0.71–3.23)	1.45 (0.68–3.08)
Any Surgical Infection	0.92 (0.47–1.79)	0.92 (0.47–1.81)
Any Reoperation	0.48 (0.18–1.30)	0.48 (0.18–1.33)
Any Readmission	1.04 (0.68–1.59)	1.08 (0.70–1.84)
Readmission Related to Operation	1.25 (0.74–2.11)	1.25 (0.74–2.12)
Bleeding Requiring Transfusion	0.84 (0.11–6.14)	0.54 (0.05–6.03)
Death of Patient	4.05 (0.49–33.6)	2.60 (0.24–28.05)

*Adjusted for age (days), sex, ASA classification, weight, presence of bleeding or hematologic disorder, pulmonary comorbidity, liver/biliary/pancreas comorbidity, cardiac comorbidity, renal comorbidity, and weight loss or failure to thrive comorbidity

**Table 4:** Linear and Multivariate Linear Regression Models Comparing General Surgeons to Pediatric Surgeon

	Intercep	Beta coefficient	Standard error of coefficient	*p*-value	Adjusted R2
Linear Regression Model					
Operative Time	29.39	-4.92	0.56	< 0.05	–
Length of Stay	2.39	-0.04	0.28	0.90	–
Days from Operation to Discharge	1.40	-0.16	0.24	0.50	–
Multivariate Regression Model*					
Operative Time	23.71	–4.56	0.55	< 0.05	0.05
Length of Stay	2.10	–0.04	0.28	0.90	0.005
Days from Operation to Discharge	1.54	–0.19	0.24	0.44	0.006

*Adjusted for age (days), sex, ASA classification, weight, presence of bleeding or hematologic disorder, pulmonary comorbidity, liver/biliary/pancreas comorbidity, cardiac comorbidity, renal comorbidity, and weight loss or failure to thrive comorbidity

**Table 5:** Temporal Trends in Surgeon Specialty and Operative Approach by 2-Year Periods

Year group	Total cases	General surgeon (%)	Attempted laparoscopic (%)	Conversion rate (%)
2012–2013	3,077	2.8	20.1	6.1
2014–2015	3,587	5.5	32.5	3.4
2016–2017	4,328	6.0	27.9	6.4
2018–2020	7,461	2.5	20.4	5.5
Overall	18,453	4.0	24.5	5.3

## Discussion

This study utilized the NSQIP-P database to compare the 30-day surgical outcomes between pediatric patients who underwent pyloromyotomy by either a GS or PS within NSQIP-P participating institutions, which are primarily pediatric-focused. Outcomes for patients were largely similar between the two groups even after controlling several patient demographic factors via multivariable logistic regression.

Previous literature examining post-operative complications of pediatric patients undergoing operations by GS or PS has shown mixed results [9–15]. Some studies report increased morbidity after pyloromyotomies with GS compared to PS [9, 12, 13], while others maintain that hospital and surgeon volume, not specialty, dictates outcomes, including cost considerations [14, 15]. Our findings of no significant difference in adjusted odds ratios (e.g., postoperative complications OR 0.92, 95% CI 0.47–1.81; Table 3) align with volume-based studies but contrast with specialty-focused reports. For example, similar to Ednie et al. (2017) on Canadian regional outcome variations [16], our NSQIP-P data shows comparable results in pediatric-focused settings. However, national trends in Bakir et al. (2025) indicate shifts toward PS and laparoscopy [17], In our cohort, time-trend analysis by 2-year periods showed only modest variation in both GS proportion and laparoscopic use, with transient peaks in the middle years (Table 5). Compared to Donda et al. (2019), our cohort also had a higher proportion of white patients (73% vs. 50%), further suggesting selection bias [18].

With IHPS continuing to be one of the most common diagnoses requiring operative intervention, these results suggest the operation could safely be performed outside specialized centers, though further validation is needed.

This study found no difference in patient outcomes when comparing GS and PS. Overall, the percentages of post-operative complications examined were low, with rates of readmission, surgical site infection, reoperation, and death found to be 3%, 1%, 1%, and 0.04%, respectively. No difference was seen even after controlling for several demographic characteristics. While GS were found to have a statistically significant shorter operative time and length of stay, neither of these represented clinically significant differences. This may be also explained by an increased likelihood of trainee involvement, which are more common at larger tertiary academic centers where PS are concentrated.

Additionally, GS were more likely to perform the operation via an open approach (Adjusted OR 1.24, 95% CI 1.04–1.49), consistent with Table 2 (GS 75.0% open vs. PS 67.3%). This likely reflects GS’s greater familiarity with open techniques in infants due to limited pediatric laparoscopic training, as laparoscopic pyloromyotomy requires specialized skills more commonly developed in pediatric surgery fellowships.

The conclusions of this study come with several important limitations. As with all retrospective studies, the information gathered from the charts was entered for clinical, not research purposes, which likely results in missing data points or erroneous information. A significant limitation is the potential misclassification bias in the NSQIP-P database. For example, it is unclear whether surgeons classified as GS truly lack pediatric surgery fellowship training, as some PS may be recorded as GS, particularly in pediatric hospitals. This misclassification could homogenize outcomes between general and PS, potentially biasing findings toward the null hypothesis of no difference. Additionally, GS in the NSQIP-P database are defined as those working at specific types of facilities (e.g., freestanding general acute care children’s hospitals, children’s hospitals within larger hospitals, specialty children’s hospitals, or general acute care hospitals with a pediatric wing), which may not be generalizable to all GS in the community setting. A future study comparing pyloromyotomies performed by GS in the NSQIP Adult database with those performed by PS in the NSQIP-P database could provide a more accurate comparison of outcomes between these groups. There is no information in the NSQIP-P database pertaining to the operating surgeons’ case volumes related to pyloromyotomies or, more generally, operative experience with pediatric populations. It is possible that the GS performing these cases only choose to perform them because of previous experience and familiarity. The study is also limited by the extreme difference between the group sizes, as the number of surgeons classified as PS far outnumbered those classified as GS (731 GS vs. 17,722 PS). Due to this imbalance, small effect sizes may be undetected, and rare events (e.g., mortality 0.04%) produced wide confidence intervals, reducing reliability. Hospital clustering was not adjusted statistically due to lack of hospital identifiers in the data. Propensity score matching or weighting was not performed due to data constraints, though multivariable regression was used to adjust for known confounders. Additionally, the low incidence of operative complications and preexisting medical conditions may have obscured any true differences between the groups in this analysis. The data in the NSQIP-P database are derived from participating hospitals, which are primarily pediatric-focused, and may not be generalizable to all adult or non-pediatric surgery hospitals. Lastly, the study is limited by the 30-day follow-up available in the NSQIP-P database. While complications more than 30 days out after a pyloromyotomy are rare, any complications that arise after this time period could not be assessed.

## Conclusion

Within NSQIP-P pediatric-focused institutions, pyloromyotomies by GS showed no difference in postoperative complications or mortality compared to PS, despite GS preferring an open approach. These findings apply only to administratively-coded surgeon categories in participating institutions and do not inform broader policy on surgical scope due to database limitations, including potential misclassification of surgeon specialty and lack of data on training or case volume. Further validation is needed, potentially through comparisons with NSQIP Adult data to assess GS outcomes in community settings.

## Data Availability

No datasets were generated or analysed during the current study.

## Abbreviations

IHPS: Infantile hypertrophic pyloric stenosis
NSQIP-P: National surgical quality improvement program, pediatric
CPT: Current procedural terminology
ASA: American society of anesthesiologists
